# Supplementary figures and images for: Analysis of Serial Neuroblastoma PDX Passages in Mice Allows the Identification of New Mediators of Neuroblastoma Aggressiveness
Source: Int J Mol Sci. 2023 Jan 13;24(2):1590. doi: 10.3390/ijms24021590 (PMC9866967; doi:10.3390/ijms24021590)

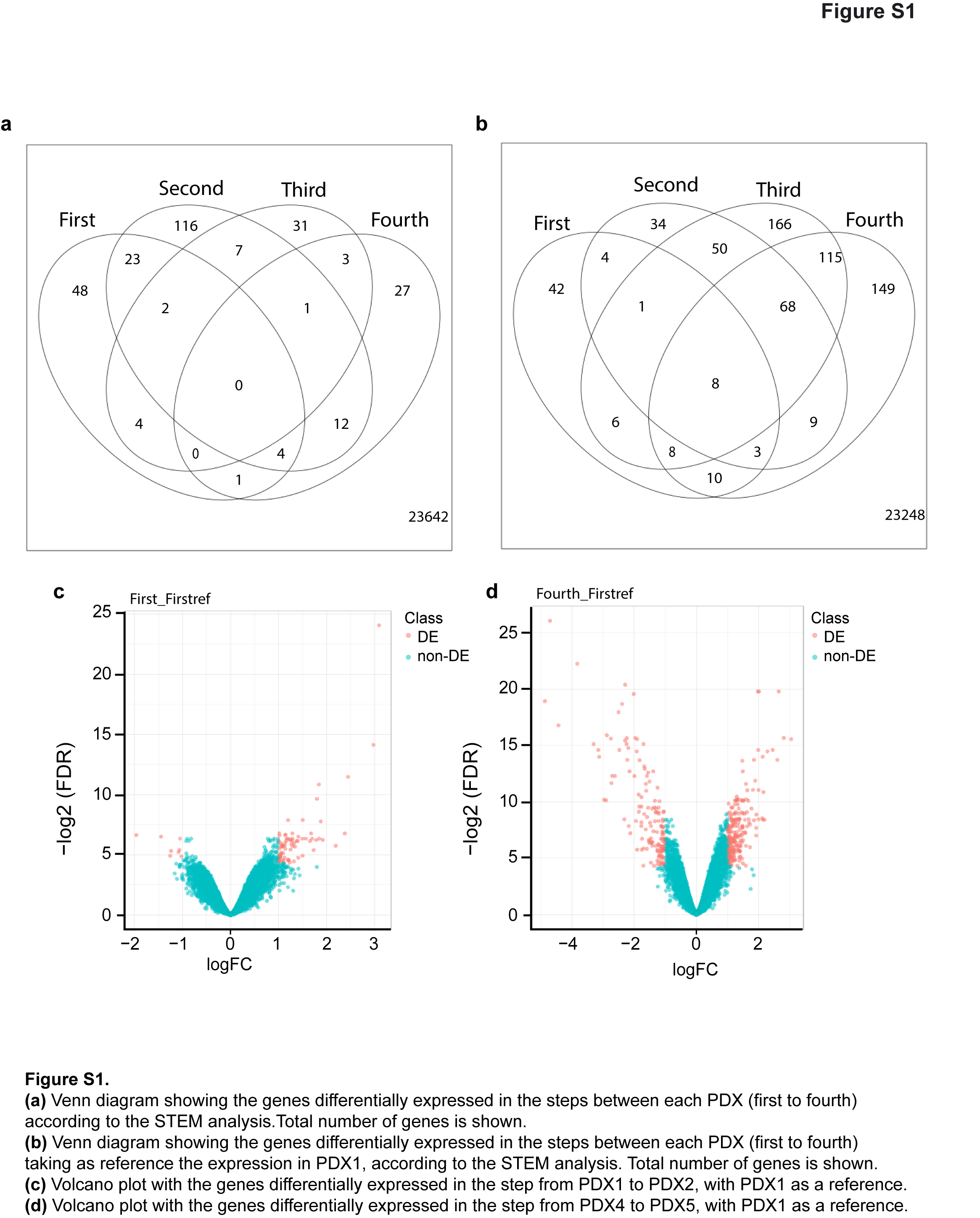

Supplement: Supplementary file 1 [file ijms-24-01590-s001.zip › FigureS1_V3.tif]

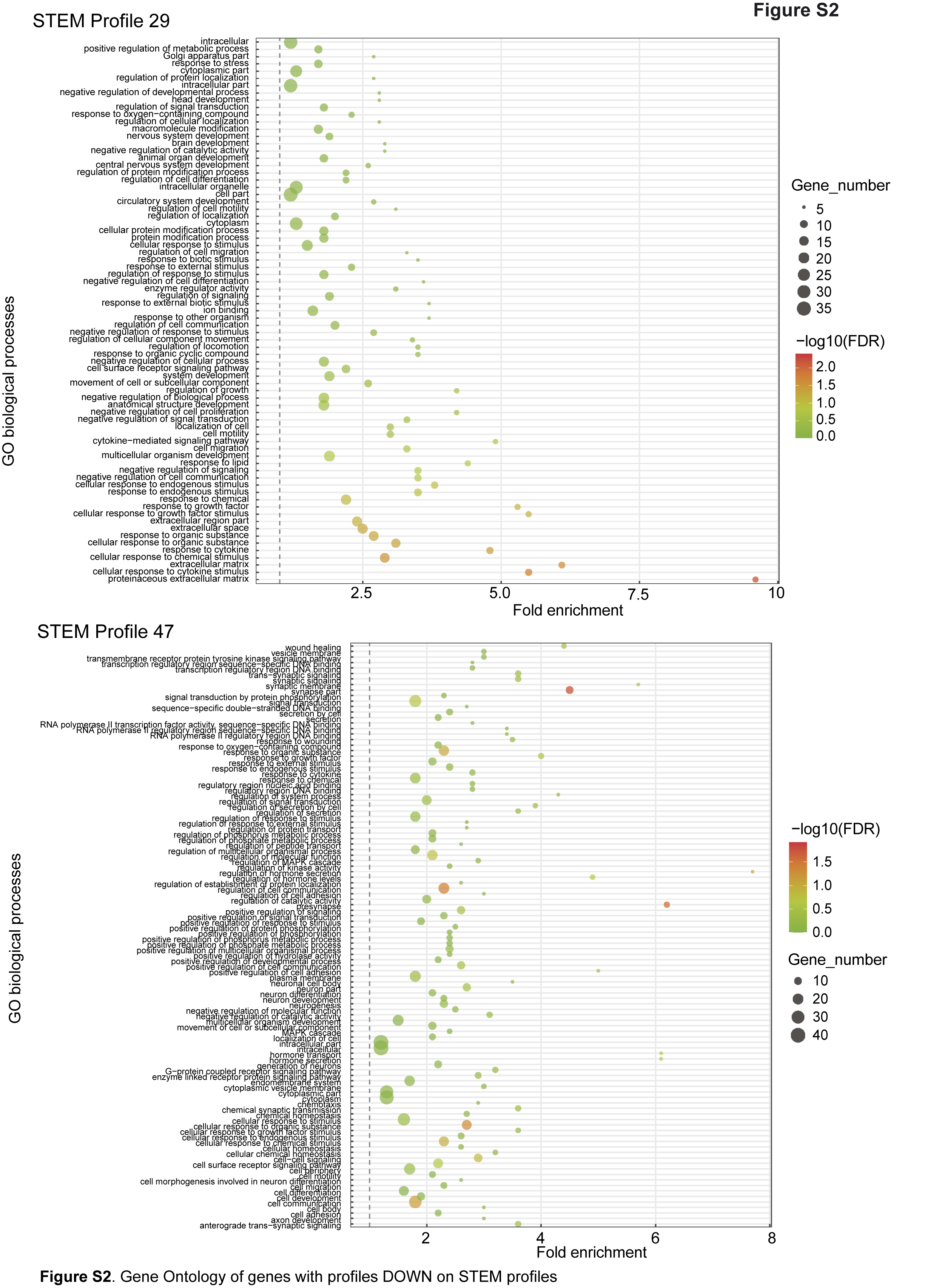

Supplement: Supplementary file 1 [file ijms-24-01590-s001.zip › FigureS2_final_V1.tif]

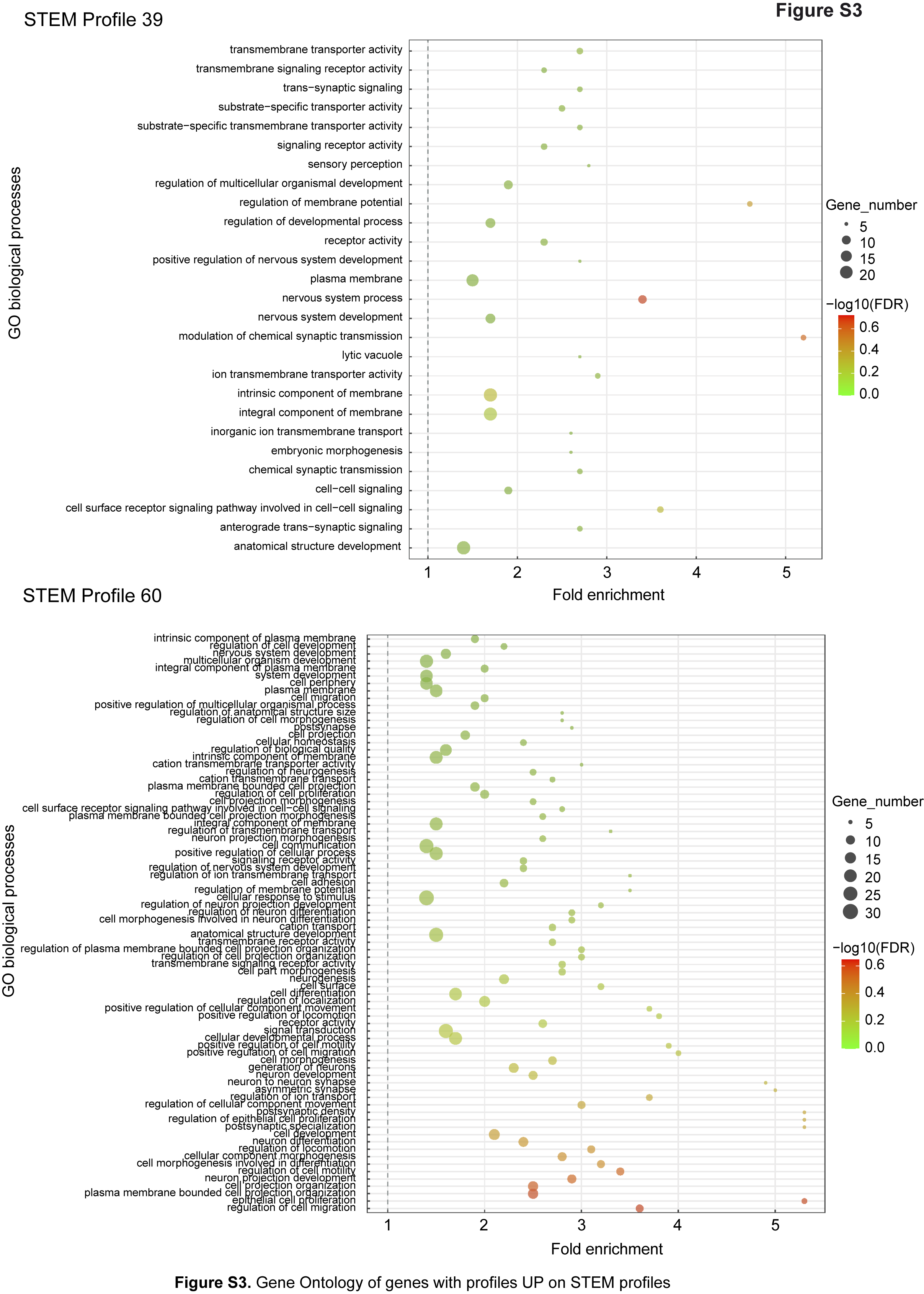

Supplement: Supplementary file 1 [file ijms-24-01590-s001.zip › FigureS3_V3.tif]

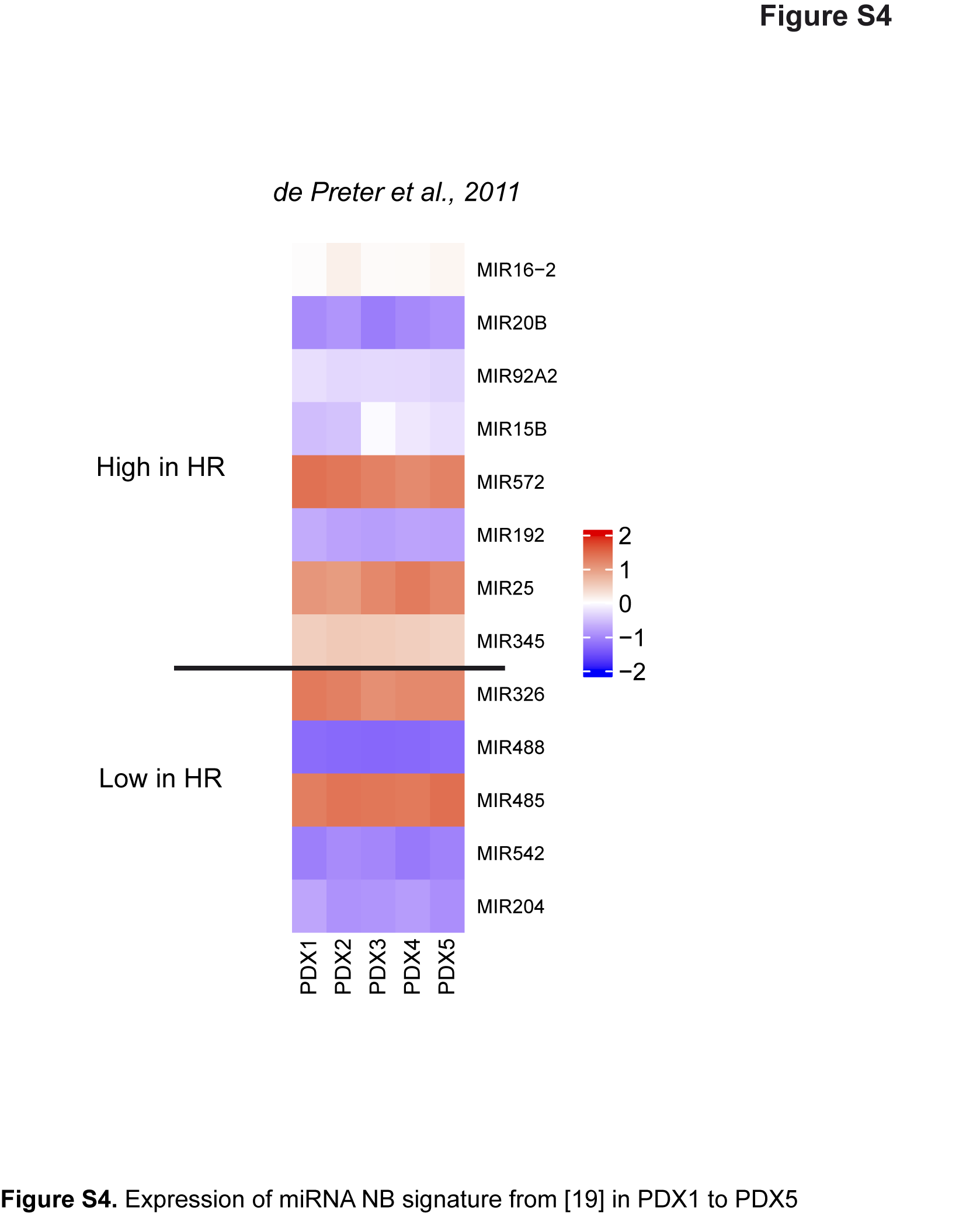

Supplement: Supplementary file 1 [file ijms-24-01590-s001.zip › FigureS4_V2.tif]

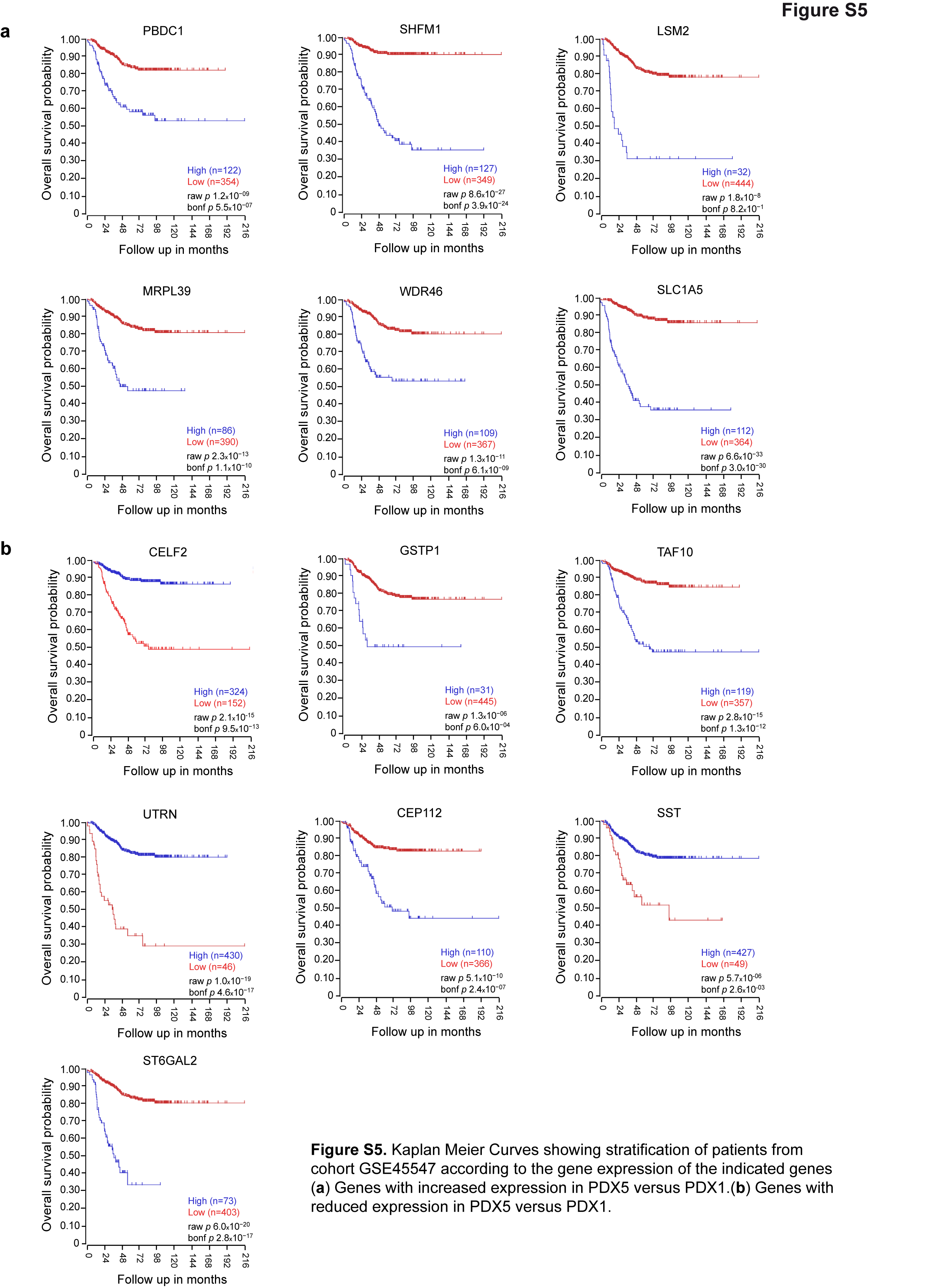

Supplement: Supplementary file 1 [file ijms-24-01590-s001.zip › FigureS5_V3.tif]

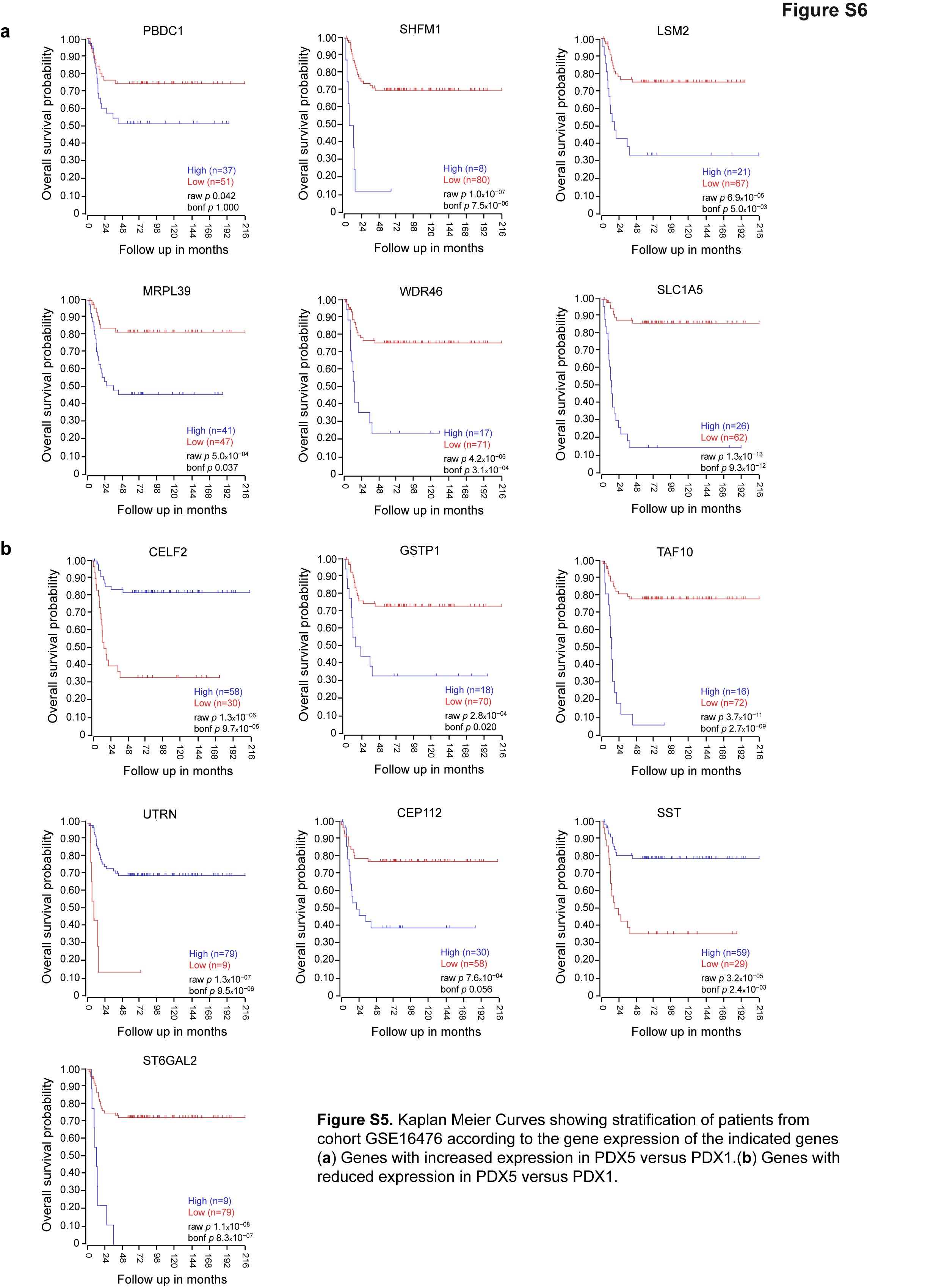

Supplement: Supplementary file 1 [file ijms-24-01590-s001.zip › FigureS6_V2.tif]
